# AWARE: Anaesthesia trainee wellbeing at respective educational institutions: A national prospective study of burnout, depressive symptoms and occupational stress

**DOI:** 10.4102/sajpsychiatry.v32i0.2630

**Published:** 2026-07-28

**Authors:** Ryan van der Merwe, Thenjiwe Hlongwane, Patricia Brown, Palesa Mogane

**Affiliations:** 1 Department of Anaesthesia, Faculty of Clinical Medicine, University of the Witwatersrand, Johannesburg, South Africa; 2 Department of Anaesthesia, Chris Hani Baragwanath Academic Hospital, Johannesburg, South Africa; 3 Private Practice, Netcare Linksfield Hospital, Johannesburg, South Africa; 4 Private Practice, Sandton Medpark, Johannesburg, South Africa

**Keywords:** burnout, depression, anaesthesiologists, South Africa, public hospitals

## Abstract

**Background:**

Anaesthesia trainees are at increased risk of burnout and depression – with occupational stress recognised as a trigger for both. Previous South African single-site studies have assessed these outcomes separately, but not within the same study.

**Aim:**

This study aimed to describe a composite mental well-being profile of South African anaesthesia trainees, focusing on burnout, depression screening, occupational stress and associated risk factors.

**Setting:**

The study was conducted among South African anaesthesia trainees from nine university-affiliated public teaching hospitals.

**Methods:**

Participants were recruited via convenience sampling through engagement with study representatives. Burnout, depression screening and occupational stress were measured using the abridged Maslach Burnout Inventory-Human Services Survey for Medical Personnel, the Harvard Department of Psychiatry National Depression Screening Day Scale and the Effort–Reward Imbalance questionnaire. The response rate was 37%.

**Results:**

Of the 194 trainees, 54 (28%) met burnout criteria, 88 (45%) screened positive for depression and 144 (74%) demonstrated chronic occupational stress. Forty-one (21%) trainees simultaneously met criteria for all three study outcomes, representing a distinctly vulnerable subcohort. Risk factors included: previous and/or current treatment for psychiatric illness, registrars in their second year of training and self-prescribing anxiolytics and antidepressants.

**Conclusion:**

Burnout rates in this population closely approximate international estimates; however, both depressive symptoms and occupational stress prevalence exceed international figures.

**Contribution:**

This first-of-its-kind study provided a composite insight into the mental well-being profile of South African anaesthesia trainees, identifying a bigger cohort at risk of burnout and/or depression in the near future.

## Introduction

The discipline of anaesthesia requires its providers to assume responsibility for overseeing physiological homeostasis in an unconscious patient undergoing a procedure.^1^ This demands considerable vigilance and rapid clinical decision-making, often during intraoperative crises.^2^

Therefore, anaesthetists experience high levels of perceived stress, and such chronic exposure may have deleterious effects on their mental wellbeing.^2^ Within the field of anaesthesiology, trainees have been identified as a particularly vulnerable group given their long working hours and necessity to navigate both their clinical workload and academic obligations.^3^ A 2017 report from the Royal College of Anaesthetists found that 61% of anaesthesia trainees felt their job negatively affected their mental wellbeing.^4^

The concept of mental wellbeing is broad and complex.^5^ Over the last 40 years, two components in the field have been the subject of ever-increasing research: burnout and depression and their effects on workplace performance.^6^ Such research has been the catalyst for introducing workplace wellbeing programmes to improve wellbeing in a workforce.^7^

Burnout is defined in the 11th revision of the International Classification of Diseases (ICD-11) as a ‘syndrome conceptualised as resulting from chronic workplace stress that has not been successfully managed’.^8^ Its formal incorporation into a classification system was only done in 2021; however, burnout appeared in the literature in the 1970s, accumulating over 15 measurement tools. Comparison between studies is limited because of considerable study heterogeneity.^9^ In a 2018 systematic review of burnout among physicians (*n* = 109 628) across 45 countries, the Maslach Burnout Inventory (MBI) was used in 53% of responses (*n* = 58 181), highlighting its widespread acceptability.^9^ The MBI is a measuring tool used to quantify burnout in a population comprising three sub-scales: emotional exhaustion (EE), depersonalisation (DP) and personal accomplishment (PA).^10^ It allows the researcher to determine individuals considered at high risk for burnout.^11^ Research conducted in the 1990s found that decreased job performance results from a dissatisfied workforce; and that a distressed workforce, that is one at risk of burnout, is more likely to be dissatisfied.^12^

A second focus of mental wellbeing research has been depression. Depression, as classified in the American Psychiatric Association’s *Diagnostic and Statistical Manual of Mental Disorders*, Fifth Edition, Text Revision (DSM-5-TR), forms a spectrum of mood disorders which all demonstrate a depressed mood with anhedonic features, accompanied by somatic and cognitive alterations.^13^ This disruption must result in functional impairment to meet the definition. Screening for depressive symptoms can be performed using the Harvard Department of Psychiatry National Depression Screening Day Scale (HANDS).^14^ A positive screen suggests the presence of a major depressive disorder with a test sensitivity of 95% and specificity of 60%.^15^

Anaesthesia providers have been the focus of numerous burnout studies.^16^ A 2018 French study found a burnout prevalence of 24.4% among their anaesthetists.^17^ Three American studies, conducted in 2013, 2020 and 2022, found anaesthesia trainee burnout prevalences of 41%, 13.8% and 24%, respectively.^18,19,20^ The 2013 and 2022 studies also reported the prevalence of depression among anaesthesia trainees to be 22% and 15%, respectively, with the 2013 cohort demonstrating a 2% prevalence of suicidal ideation, double the general population risk.^18,20^

South African studies report varying degrees of burnout among anaesthetists. A 2015 study found a 25% burnout prevalence among Johannesburg state-sector anaesthetists, and an 8% prevalence among their private-sector colleagues.^21^ In contrast, a 2020 study conducted in Cape Town state-sector anaesthetists reported a burnout prevalence of only 4%.^22^

Multiple studies quote female gender, increased workload, excessive alcohol consumption (> 5 units/week) and previous psychiatric diagnosis as significant risk factors implicated in the development of burnout and depression.^17,18,20,21,22^

Burnout and depression are considered late-occurring events with widespread consequences, such as increased risk of early mortality, frequent absenteeism, diminished productivity and organisational financial losses.^23^ There is consensus that chronic stress exposure overwhelms coping mechanisms, resulting in maladaptive behaviour with ensuing burnout and depression.^24^

Occupational stress has been the subject of research since the 1980s and was initially attributed to either extrinsic or intrinsic factors.^25^ Social reciprocity in the workplace, as the basis for occupational stress, was only considered in the late 1990s, where expended workplace efforts should be met with appropriate reciprocal rewards.^25^ An imbalance would result in occupational stress. The Effort-Reward Imbalance questionnaire (ERI) was borne out of this theoretical research. It is a widely accepted and validated tool to measure occupational stress and has been used in 41 studies (*n* = 53 642 participants).^26^ In nine of these 18 studies, reporting ERI rates > 1 (indicative of a workplace imbalance suggestive of occupational stress), researchers found rates between 20% and 80.7%.^26^ Prompt identification of chronic occupational stress in a workforce provides insight into a vulnerable cohort at risk of future development of burnout and/or depression if the stress goes unmanaged.

Limitations identified in previous South African studies include single-study-site populations and measuring only one psychological outcome of interest. Previous studies were conducted before the coronavirus disease 2019 (COVID-19) pandemic, which significantly affected the global healthcare systems between 2020 and 2023. The COVID-19 pandemic resulted in a considerable increase in psychological distress experienced by healthcare workers^27^ and therefore, may have impacted mental wellbeing among anaesthetists.

This first-of-its-kind study aimed to describe a composite mental wellbeing profile of South African anaesthesia trainees, with a particular focus on burnout, depressive symptom screening and occupational stress. The study also aimed to ascertain their respective risk factor profiles.

## Research methods and design

### Study design

This study employed a contextual, prospective, cross-sectional design by distributing three questionnaires: the abridged Maslach Burnout Inventory-Human Services Survey for Medical Practitioners (aMBI-HSS [MP]), HANDS and ERI. These are tools that screen for burnout, depressive symptoms and occupational stress, respectively. The study was conducted between 05 March 2024 and 11 September 2024.

### Study setting

The study was conducted across nine South African university-affiliated teaching hospitals, which represent the accredited postgraduate anaesthesia training sites. These hospitals are tertiary- and quaternary-level facilities, encompassing various surgical disciplines.

The study population comprised anaesthesia trainees defined either as: (1) registrars enrolled in a formal specialist training programme or (2) medical officers in contractual posts intending to apply for registrar training. In South Africa, anaesthesia registrar training is a 4-year programme supervised by university-affiliated departments and aligned with the Colleges of Medicine of South Africa (CMSA) curriculum.

Participants were obtained through convenience sampling. At each site, an authorised anaesthesia registrar representative distributed the REDCap® online questionnaire link via departmental email lists and messaging platforms to all eligible trainees. Reminders were sent twice monthly for the duration of the study. Participation was voluntary, and study eligibility was confirmed within the survey.

### Data collection

Data were collected using the REDCap® online questionnaire. The study questionnaire comprised the following sections: demographic details, aMBI-HSS (MP), HANDS and the shortened ERI questionnaires. Demographic variables were presented as multiple-choice, and the aMBI-HSS (MP), HANDS and ERI questionnaires as Likert-scales. Data stored on REDCap® was exported into a Microsoft Excel® spreadsheet. A total of 238 responses were received. Thirty-three records were incomplete (missing demographic information [11]; incomplete aMBI-HSS [MP] data [14]; incomplete HANDS data [5]; incomplete ERI data [3]), and 11 records did not meet the study inclusion criteria. After exclusion of these 44 records, 194 complete records were used for the final data analysis. The calculated response rate was 37% (194 complete responses/529 possible training posts).

### Data analysis

Burnout is defined as high scores in the EE subscale (≥ 27 points), DP subscale (≥ 10 points) and a concomitant low score in the PA subscale (≤ 33 points) using the aMBI.^10^ Participants were classified as high burnout if they met all three criteria; otherwise, they were classified as low-moderate burnout. A positive screening on the HANDS questionnaire is defined as a score equal to nine or more (out of 30), which indicates a high likelihood of depression and warrants further diagnostic investigation. Using the ERI as a measure for occupational stress: when the ERI = 1, there is balance in the workplace. Where there is an occupational imbalance, indicated by an ERI < 1, the imbalance arises from excessive rewards reaped for the effort expended. When the ERI > 1, the imbalance comes from excessive expended effort outweighing the rewards, which is the metric of interest for occupational stress. A composite outcome was created, defined as participants screening positive for burnout, depressive symptoms and occupational stress concurrently. This was termed a ‘positive triple screen’ and analysed for associated risk factors.

Data were analysed using STATA v.18. Descriptive data analysis included summaries of the demographics, the aMBI-HSS (MP), HANDS and ERI results using frequencies and percentages. Scale reliability coefficients were reported for each questionnaire and each question item. The scale reliability coefficient for the aMBI-HSS (MP), HANDS and ERI questionnaire in this study was 0.87, 0.89 and 0.81, respectively. A scale reliability coefficient (Cronbach’s alpha) > 0.7 is acceptable and demonstrates high internal consistency of the measuring tool.^28^ Normality was assessed using the Shapiro–Wilk test, with a *p*-value < 0.05 indicating normal distribution. Inferential data analysis sought to determine any association between demographic variables and the scores of the screening tools. A comparison between the registrar and medical officer categories was done using the Chi-square test. In contrast, the Mann–Whitney U-test assessed the statistical significance of the demographic variables and questionnaire scores. A *p*-value of < 0.05 was considered statistically significant. The Chi-square test and univariate logistic regression analysis evaluated factors associated with the study outcomes.

### Ethical considerations

This study was jointly approved by the University of the Witwatersrand (Wits) Human Research Ethics Committee (Medical), certificate number M230705, and the University of Pretoria Faculty of Health Sciences Research Ethics Committee, Protocol 118/2024. Other study sites agreed to accept the ethical approval granted by the University of the Witwatersrand. Consent to participate was obtained using an online form before proceeding to the study questionnaire. A distress protocol was provided to participants via various contact information for resources offering psychological support after the questionnaire was completed. Data captured using the REDCap® platform contained no participant identifiers to ensure participant anonymity. Only the principal researcher and co-supervisors had access to the password-protected raw data.

## Results

### Demographics

One hundred and ninety-four surveys (out of a possible 529 trainee posts across South Africa) were collected and analysed – yielding a 36.7% response rate. Seventy-eight per cent (*n* = 152) of the responses were from registrars, and 22% (*n* = 42) were from medical officers. Wits trainees comprised 53% of the responses (*n* = 103), and the other eight academic circuits comprised the remaining 47% (*n* = 91). Two-thirds of responses were received from female trainees. Table 1 shows a summary of the study demographics. Self-prescribing was reported in 68% of the respondents. Thirty-six per cent of trainees (*n* = 71) had received treatment for anxiety and/or depression in the past, with 25% (*n* = 50) currently receiving ongoing treatment for the above psychological conditions.

**TABLE 1:** Demographic summary of the study participants (*N* = 194).

Demographic variable	Overall	
	*n*	%
**Academic circuit**		
University of the Witwatersrand	103	53.1
University of Pretoria	18	9.3
Stellenbosch University	15	7.7
University of the Free State	14	7.2
University of Cape Town	13	6.7
University of KwaZulu-Natal	11	5.7
Walter Sisulu University	11	5.7
Sefako Makgatho Health Sciences University	7	3.6
Limpopo University	2	1.0
**Career designation**		
Medical officer	42	21.7
Registrar	152	78.3
**Registrar year of study (*N* = 152)**		
First	40	26.3
Second	41	27.0
Third	44	28.9
Fourth or more	27	17.8
**Gender**		
Female	129	66.5
Male	65	33.3
**Age (years)**		
< 30	49	25.3
30–33	86	44.3
> 33	59	30.4
**Relationship status**		
Single or divorced	50	25.8
Committed relationship or married	144	74.2
**Parental status**		
No	126	65.0
Yes	68	35.0
**Highest qualification**		
Bachelor of Medicine/Bachelor of Surgery (MBBCh/MBChB)	11	5.7
Diploma in Anaesthesiology (South Africa) (DA [SA])	21	10.8
Fellow of the College of Anaesthesiologists of South Africa (Part 1 or Part 2) (FCA [1 or 2])	162	83.5
**Years in clinical medicine**		
3–5	41	21.1
6–8	91	46.9
9–11	48	24.7
> 11	14	7.2
**Within 3 months of exams?**		
No	156	80.4
Yes	38	19.6
**Completed Master of Medicine (MMed)? (*N* = 152)**		
No	123	80.9
Yes	29	19.1
**Smoking status**		
Non-smoker	184	94.9
Smoker	10	5.1
**Alcohol consumption**		
None	79	40.7
Yes:	115	59.3
< 5 units per week	104	53.6
> 5 units per week	11	5.7
**Recreational drug use**		
No	186	95.9
Yes	8	4.1
**Seen a general practitioner?**		
No	120	61.9
Yes:	74	38.1
In the last year	30	15.4
More than 1 year ago	44	22.7
**Self-prescribed in the last year**		
No	63	32.5
Yes:	131	67.5
Antibiotics	65	33.5
Non-opioid analgesics	50	25.7
Proton pump inhibitors	31	16.0
Oral contraceptive	30	15.5
Sleeping tablets	22	11.3
Anxiolytics	20	10.3
Anti-depressants	19	9.8
Miscellaneous	13	6.7
Opioid analgesics	11	5.7
**Previous treatment (medication or therapy) for anxiety ± depression**		
No	123	63.4
Yes	71	36.6
**Currently receiving treatment (medication or therapy) for anxiety ± depression**		
No	144	74.2
Yes	50	25.7

### Burnout

The overall prevalence of high risk of burnout in this study population was 28% (*n* = 54) (Table 2). The breakdown of the aMBI-HSS (MP) sub-scales between the registrar and medical officer group is shown in Table 2. Assessment of the sub-scales of the aMBI-HSS (MP) in Table 2 demonstrated a greater percentage of medical officers reporting low PA (45%) versus registrars (23%) which was statistically significant (*p*-value = 0.010). High risk of burnout was associated with the following statistically significant risk factors: junior trainees without any additional anaesthesia qualifications (such as a DA or FCA 1), current (OR 2.8; CI: 1.4–5.4, *p*-value = 0.004) or previous treatment for anxiety and/or depression (OR 2.2; CI: 1.2–41, *p*-value = 0.017) (Table 3). A significant observation was that none of the trainees who possessed a Diploma in Anaesthesia (DA) qualification screened positive for burnout (*p*-value = 0.002).

**TABLE 2:** Study outcome comparison – registrars versus medical officers.

Domain	Registrars (%) (*n* = 152)	Medical officers (%) (*n* = 42)	Overall (%) (*n* = 194)	*p*-value
**Burnout**				
Emotional exhaustion				0.143
High	105 (69.1)	26 (61.9)	131 (67.5)	-
Moderate	23 (15.1)	4 (9.5)	27 (13.9)	-
Low	24 (15.8)	12 (28.6)	36 (18.6)	-
Median (IQR)	30.6 (23.4–41.4)	30.6 (18–37.8)	30.6 (23.4–39.6)	0.335
Range	5.4–54	9–52.2	5.4–54	-
Depersonalisation				0.562
High	99 (65.1)	24 (57.1)	123 (63.4)	-
Moderate	29 (19.1)	11 (26.2)	40 (20.6)	-
Low	24 (15.8)	7 (16.7)	31 (16.0)	-
Median (IQR)	15.0 (6.7–20.0)	11.7 (6.7–18.4)	13.36 (6.68–20.04)	0.243
Range	0–30.1	0–26.72	0–30.1	-
Personal accomplishment				0.010*
High	72.0 (47.4)	11.0 (26.2)	83.0 (42.8)	-
Moderate	45.0 (29.6)	12.0 (28.6)	57.0 (29.4)	-
Low	35.0 (23.0)	19.0 (45.2)	54.0 (27.8)	-
Median (IQR)	34.0 (27.0–38.0)	38.0 (32.0–42.0)	36.0 (28.0–40.0)	0.002*
Range	8.0–48.0	12.0–48.0	8.0–48.0	-
**Depression**				-
Positive screen	70 (46.1)	18 (42.9)	88 (45.4)	0.730
Median (IQR)	7.5 (4–12)	7.5 (4–11)	7.5 (4–12)	0.580
Range	0–30	0–25	0–30	-
**Occupational stress**				
ER > 1	115 (75.7)	29 (69.05)	144 (74.2)	0.420
Median (IQR)	1.2 (1.0–1.5)	1.1 (1.0–1.4)	1.2 (1.0–1.5)	0.081
Range	0.6–3.5	0.7–3.5	0.6–3. 5	-

ER, effort reward ratio; IQR, interquartile range.

* , *p*-value < 0.05 - statistically significant.

**TABLE 3:** Risk factors associated with each study outcome.

Variable	Positive screen		*p*-value	Univariate analysis		*p*-value
	*n*	%		OR	95% CI	
**Burnout**						
Highest qualification						
MBBCh/MBChB	6	54.60	0.002	-	-	-
DA(SA)	0	-		-	-	-
FCA (1 or 2)	48	29.60		-	-	-
Current treatment for anxiety +/– depression						
No	32	22.20	0.003	1*	-	-
Yes	22	44.00		2.8	1.4–5.4	0.004
Previous treatment for anxiety +/– depression						
No	27	22.00	0.016	1*	-	-
Yes	27	38.00		2.2	1.2–4.1	0.017
**Depressive symptoms**						
Highest qualification						
MBBCh/ MBChB	9	81.80	0.016	1*	-	-
DA(SA)	6	28.60		0.1	0.0–0.5	0.008
FCA (1 or 2)	73	42.70		0.2	0.04–0.9	0.033
Current registrar year of study						
First	13	32.50	0.020	1*	-	-
Second	27	65.90		4.0	1.5–10.1	0.003
Third	19	43.20		1.6	0.6–3.8	0.315
Fourth or more	11	40.70		1.4	0.5–3.9	0.491
Self-prescribed anxiolytics						
No	73	42.00	0.005	1*	-	-
Yes	15	75.00		4.2	1.4–11.9	0.008
Self-prescribed anti-depressants						
No	72	42.10	0.013	1*	-	-
Yes	16	69.60		3.1	1.2–8.0	0.017
Current treatment for anxiety +/– depression						
No	48	33.30	< 0.001	1*	-	-
Yes	40	80.00		3.2	3.7–17.4	< 0.001
Previous treatment for anxiety +/– depression						
No	44	35.80	< 0.001	1*	-	-
Yes	44	62.00		2.9	1.6–5.4	< 0.001
**Occupational stress**						
Current registrar year of study						
First	24	60.00	0.046	1*		
Second	35	85.40		3.8	1.3–11.4	0.013
Third	34	77.30		2.3	0.9–5.8	0.090
Fourth or more	22	84.50		2.9	0.9–9.3	0.915
Current treatment for anxiety +/– depression						
No	101	70.10	0.027	1*	-	-
Yes	43	86.00		2.6	1.1–6.3	0.031
**Positive triple screen (scored positive for all 3 study outcomes concurrently)**						
Self-prescribed anti-depressants						
No	31	75.60	0.005	1*	-	-
Yes	10	24.39		3.5	1.4–8.6	0.007

DA, Diploma in Anaesthesia; FCA, fellow of the college of anaesthesiologists; SA, South Africa; CI, confidence interval; MBChB/MBBCh, Medicinae Baccalaureus, Baccalaureus Chirurgiae.

* , Denotes reference category used for odds ratio calculation.

### Depression

Forty-five per cent of respondents screened positive as high risk for depression using the HANDS tool. Ten per cent of trainees (*n* = 18) reported having suicidal ideation ‘some of the time’ or more frequently, with 1% (*n* = 2) reporting suicidal ideation ‘all of the time’. Risk factors associated with a positive HANDS screen included: Registrars in their second year of training (OR 4.0; CI: 1.5–10.1, *p*-value = 0.03) and current (OR 3.2; CI: 2.7–17.4, *p*-value < 0.001) or previous (OR 2.9; CI: 1.6–5.4, *p*-value < 0.001) treatment for anxiety and/or depression. Table 3 highlights that those who self-prescribed anxiolytics (OR 4.2; CI: 1.4–11.9, *p*-value = 0.008) and anti-depressants (OR 3.1; CI: 1.2–8.0, *p*-value = 0.017) were statistically significant additional risk factors for a positive HANDS screen.

Self-reported substance use among trainees in this study included: alcohol consumption in 59%, smoking in 5% and recreational drug use in 5% of trainees. Self-prescription of medication was reported in two-thirds of trainees. Of those who self-prescribed medication, sleeping tablets were the most frequently reported (11%), followed by anxiolytics and anti-depressants (both 10%), while 6% reported using opioid analgesics.

### Occupational stress

The prevalence of occupational stress in this study was 74% (*n* = 144). Statistically significant risk factors identified in association with occupational stress include: registrars in their second year of training (OR 3.8; CI: 1.3–11.4, *p*-value = 0.013) and currently receiving treatment for anxiety and/or depression (OR 2.6; CI: 1.1–6.3, *p*-value = 0.031) (Table 3).

### Interplay between study outcomes

**FIGURE 1:**
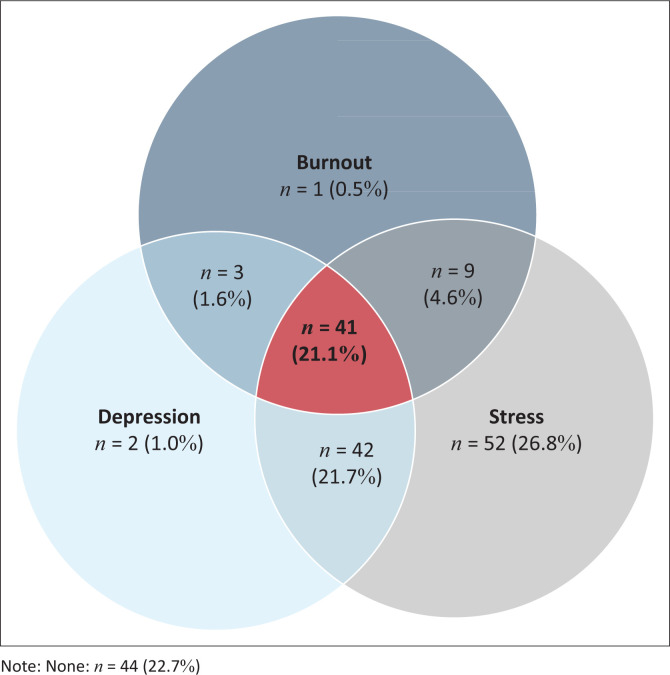
Interplay between study outcomes (*N* = 194).

Figure 1 shows the interaction between the three study outcomes and the number of respondents who met the definition for the composite study outcome referred to as a ‘positive triple screen’. Twenty-one per cent of trainees (*n* = 41) were classified as having a positive triple screen. Self-prescription of anti-depressants was significantly associated with a positive triple screen (OR 3.5; CI: 1.4–8.6, *p*-value = 0.007).

## Discussion

### Burnout

Burnout prevalence among anaesthesia trainees in this study was 28%. This figure is in keeping with international prevalence rates among both trainees and anaesthesiologists.^17,19,20^ Burnout in this study was only slightly increased when compared to the state-sector anaesthetists in the Wits 2015 cohort (21%). In contrast, this study cohort demonstrated a seven-fold increase in burnout among state-sector anaesthetists compared to the 2022 Cape Town cohort (4%), highlighting provincial differences in burnout within the state-sector healthcare system.^21,22^

Burnout prevalence between registrars and medical officers differed – 31% of registrars versus 17% of medical officers – although this difference was not statistically significant. The varied responsibilities and pressures associated with the registrar programme could explain why they may experience increased levels of burnout compared to their medical officer colleagues. A second contributor to the increased overall burnout prevalence could be attributed to the increased psychological distress following the COVID-19 pandemic, as outlined in the 2023 systematic review by Arias-Ulloa et al.^27^

The risk factor profile associated with developing burnout in this study cohort differed from the risk factors quoted in other studies. However, this study found that trainees currently or previously receiving treatment for anxiety and/or depression were found to be significant risk factors for the development of burnout. This particular risk factor was also significant in a 2019 burnout study among healthcare workers at a Johannesburg academic hospital.^23^

On analysis of the aMBI (HSS-MP) sub-scales, the medical officer group of trainees reported low personal accomplishment more frequently than their registrar counterparts, 45% versus 23%. An offer into a sought-after anaesthesiology postgraduate training programme may afford registrars a sense of accomplishment regarding job security and future professional prospects that their medical officers and colleagues have yet to experience. In recent years, with the enforcement of staffing moratoria (post-freezing) in an attempt to reduce healthcare expenditure, fewer available posts may also contribute to the lower levels of personal accomplishment reported among the medical officer cohort.^29^

The Diploma in Anaesthesia qualification allows the practitioner to enter the private-sector to provide anaesthesia to low-risk patients requiring low-risk surgical procedures. According to Van der Walt’s 2015 study, anaesthetists working in the private-sector experience significantly less burnout than their state-employed colleagues, 8.1% versus 21%.^21^ Having a means to leave the overburdened and under-resourced state healthcare system could provide insight into the absence of burnout in this particular group of trainees.

### Depression

Overall depression prevalence in this study was 45%, with similar rates between both trainee groups. This finding is significantly higher than in the two American studies conducted in 2013 and 2022, which demonstrated prevalence among trainees of 22% and 15%, respectively.^18,20^ However, this study’s prevalence was lower than the 54% prevalence of depression observed among doctors at a Johannesburg academic hospital (although this study included other medical specialities).^23^

Depression prevalence in the general population of South Africa from a 2021 population-weighted study was found to be 25.7%, in contrast with this study’s prevalence of 45%, which is in keeping with higher depression prevalence in healthcare workers worldwide.^30^ Suicidal ideation among South African trainees in this study (10%) closely approximates the observed rate of suicidal ideation among Australian anaesthetists (13%) in a 2017 report.^2^

Substance use among anaesthetists is inextricably linked to mental wellbeing. Substance use can occur in a multitude of behaviours among healthcare workers – risky use of legal substances, use of illegal substances and inappropriate use of scheduled medications. Self-prescribing rates observed in this study (67.5%) far exceed the 47% of Australian anaesthetists observed to be self-prescribing in a 2013 study.^31^ In comparison, a 2018 study involving South African anaesthetists found 85% used alcohol, 29% tobacco, 74% sedatives and 7% opioids (in the preceding 3 months).^31^ While opioid use between this study and the 2018 anaesthetist group was similar (6% vs 7% respectively), use of other substances in our study group appears to be underreported.^31^

Such underestimates of substance use in our study could be explained by the respondents being reluctant to disclose such sensitive information out of concern for being labelled as an ‘impaired physician’. The concept of the impaired physician emerged in 1970s, when the American Medical Association defined it as ‘the inability to practice medicine with reasonable skill and safety to patients by reason of physical or mental illness or alcoholism or drug dependency’.^32^ While this study did not aim to study the prevalence of substance use among anaesthesia trainees, it is well-documented that medical practitioners do not seek help in the same manner as the general public. There is often denial, an inability to recognise the problem or a feeling that they can manage it themselves.^33^ The findings of this study would reinforce that notion by the number of trainees who are self-prescribing psychotropic medication. Considerable stigma persists among the medical fraternity toward any practitioner deemed unfit or unsafe to practice, which may hinder the appropriate seeking of specialist psychiatric services. While the substance use prevalence rates reported in this study do not necessarily imply a similar prevalence of professional impairment, it is prudent to be aware that a proportion of such trainees may be at risk for developing such impairment in future.

### Occupational stress

Occupational stress is non-uniform and complex, highlighted by the existence of over 50 measurement tools to assess it.^25^ This study used the ERI, a widely accepted and validated tool to assess occupational stress. Anaesthesia trainees in this study reported a 74% prevalence of chronic occupational stress (ERI rate > 1), with the prevalence of medical officers marginally lower than their registrar counterparts. However, this difference was not statistically significant.

A 2018 systematic review assessing occupational stress among 53 642 healthcare workers using the ERI demonstrated that at least 20% of healthcare workers suffered from effort-reward imbalance, with the 2012 Greek subcohort demonstrating an occupational stress prevalence of 80.7%.^26,34^ This particular Greek study was conducted during the Greek government-debt crisis (2007–2014). It heralded a significant decline in the Greek gross domestic product per capita, with an efflux of well-educated professionals causing a deleterious effect on their public health system. The fiscal uncertainty could explain the disproportionately high prevalence of occupational stress experienced among Greek healthcare workers compared to other geographical locations.^35^ Of concern is that our study reports an occupational stress prevalence of similar magnitude to the 2012 Greek cohort. This could highlight a similar trend in the deterioration of the public health system that the South African public health sector is currently experiencing.^36^

In a 2024 address made by the South African Medical Research Council (SAMRC) CEO, Professor Ntobeko Ntusi, reflecting on the current state of South African healthcare, he acknowledged that the system is characterised by a severe shortage of medical personnel (a doctor-to-patient ratio of 0.9 per 1000 people) and a poor healthcare facility compliance rate (0.7%).^37^ These remarks followed the latest performance standards compliance assessment, which found drug availability and appropriate infection control measures to be serious concerns resulting in ‘poor coordination of service provision’ across a fragmented public sector.^37^ Several interventions aimed at addressing infrastructural inadequacies have been cited by South African doctors during the COVID-19 pandemic as strategies to reduce occupational stress – these include employing more staff, improving working conditions and availability of resources.^38^ Khan et al. demonstrate the negative mental health effects experienced by healthcare workers as a result of the poor working conditions in which they find themselves.^38^

### Interplay between outcomes

Research into the relationship between burnout and depression has demonstrated that while there is a strong correlation between the two entities (Pearson correlation 0.52), they are discrete entities which happen to share a common prerequisite: Occupational stress.^24,39^ Depression and burnout are considered late-occurring events where an individual’s coping strategies have become maladaptive. This study has demonstrated that nearly three-quarters (74%) of trainees are experiencing occupational stress – representative of a vulnerable cohort for the future development of potential burnout and/or depression. The interplay between the three entities in this study highlights that, while burnout prevalence is 28% and depression is 45%, 21% (*n* = 41) of trainees meet the study definition of a positive triple screen – a situation which undoubtedly requires prompt intervention. The second important finding is that self-prescription of anti-depressants was also associated with a significantly increased likelihood of screening positive for all three outcomes. While this does not imply causation, it does highlight that self-prescribing behaviour may be a potential marker of underlying psychological distress in a subgroup with unmet mental wellbeing needs.

### Recommendations

The Australian and New Zealand College of Anaesthetists (ANZCA) conducted national trainee wellbeing surveys in 2017 and 2019.^2,40^ This study serves as an index measure of composite wellbeing in South African anaesthesia trainees. Similar work at regular intervals should be undertaken in the future in order to track burnout, depressive symptoms and occupational stress trends over time. These future studies will also be able to guide and evaluate workplace interventions aimed at promoting trainee wellbeing.

### Limitations

There are several limitations to this study. As a result of the sensitive nature of the research and the associated stigma attached to mental wellbeing, response bias in the form of social desirability bias may result in underrepresentation of some of the study outcomes. Trainees already affected by burnout or depression may contribute to non-response bias, which underestimates the prevalence of these study outcomes. Most responses were from Wits trainees (53%), and registrars comprised 78% of responses. These factors introduce selection bias to the study and limit the generalisability of the results nationally. A further limitation in this respect is the difficulty in calculating true response rate as the estimated number of posts does not infer the total number of occupied posts in light of recent staffing moratoria. Methodological differences in burnout and depression research also limit the direct comparison of prevalence rates between studies. The HANDS questionnaire is only a depression screening tool. A qualified psychiatric assessment is required for diagnosis and treatment initiation. Observing risk factors associated with the various study outcomes does not infer causality.

## Conclusion

The composite view of the mental wellbeing profile of South African anaesthesia trainees in this study demonstrates an inordinate burden of occupational stress. Should this go unmanaged, the future burden of burnout and depression will exceed current estimates.

While the study burnout prevalence approximates international figures, depressive symptoms among this cohort far exceed those observed in similar American studies. This study identified a high rate of psychotropic self-prescription, which, coupled with the prevalence of the study outcomes, points to a vulnerable population attempting to address their own psychological distress, possibly for fear of workplace stigmatisation. The risk factor profile in this study also differed slightly from those quoted in international and local literature.

Interventions aimed at reducing the impact of burnout can either be classified as individual-directed (counselling, cognitive behaviour, skills training and relaxation training) or organisation-directed (work-schedule reorganisation, work shift evaluation and management skills training).^41^ A 2010 meta-analysis reviewing burnout interventions showed a measurable reduction in burnout following targeted interventions. Individual-directed interventions reduced burnout in the short term (lasting up to 6 months) while organisation-directed interventions reduced burnout up to 1 year, highlighting the need for a combined approach to reduce the burden of burnout in our setting.^41^
